# Supplementary figures and images for: Single-cell transcriptome analyses reveal critical roles of RNA splicing during leukemia progression
Source: PLoS Biol. 2023 May 2;21(5):e3002088. doi: 10.1371/journal.pbio.3002088 (PMC10154039; doi:10.1371/journal.pbio.3002088)

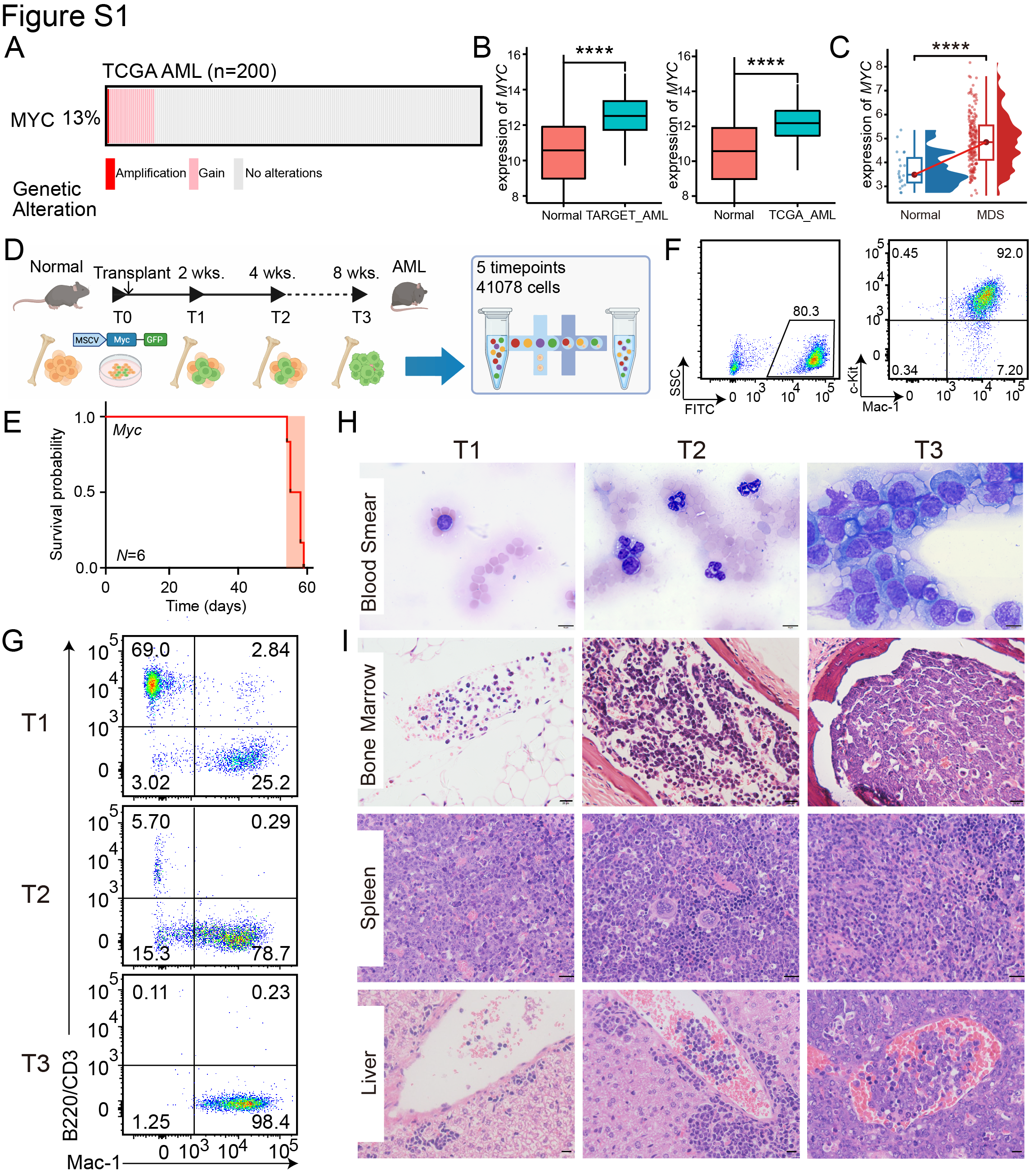

Supplement: S1 Fig — Related to Fig 1. (A) Copy number alteration of MYC in TCGA AML database. Red, Amplification; Pink, Gain; Gray, No alterations. (B) The plots showing the expression levels of MYC in normal samples and AML patients in TARGET_AML and TCGA AML cohorts. **** p.adj < 0.0001.p values were calculated by Wilcoxon test. Created with BioRender.com. (C) The plots showing the expression levels of MYC in normal samples and MDS patients generated from GSE107400. **** p.adj < 0.0001.p values were calculated by Wilcoxon test. (D) The overview of study design. (E) Kaplan–Meier survival curve of mice with Myc-GFP-induced murine AML model, (n = 6). (F) Representative flow cytometry profile of GFP-positive cells from bone marrow in T3 time point. (G) Flow cytometry profiles showing the percentage of lymphocyte and myeloid cells from peripheral blood in T1, T2, and T3 of leukemogenesis. X-axis is myeloid marker Mac-1; Y-axis is lymphocyte markers B220/CD3. (H) Blood smear of peripheral blood during T1 through T3 in Myc-GFP leukemic mouse. Scale bar: 10 μm. (I) Hematoxylin–eosin images of bone marrow (top), spleen (middle), and liver (bottom) during T1 through T3 in Myc-GFP leukemic mouse. Scale bar: 20 μm. The underlying data for S1B, S1C and S1E Fig can be found in S1 Data. (TIF) [file pbio.3002088.s001.tif]

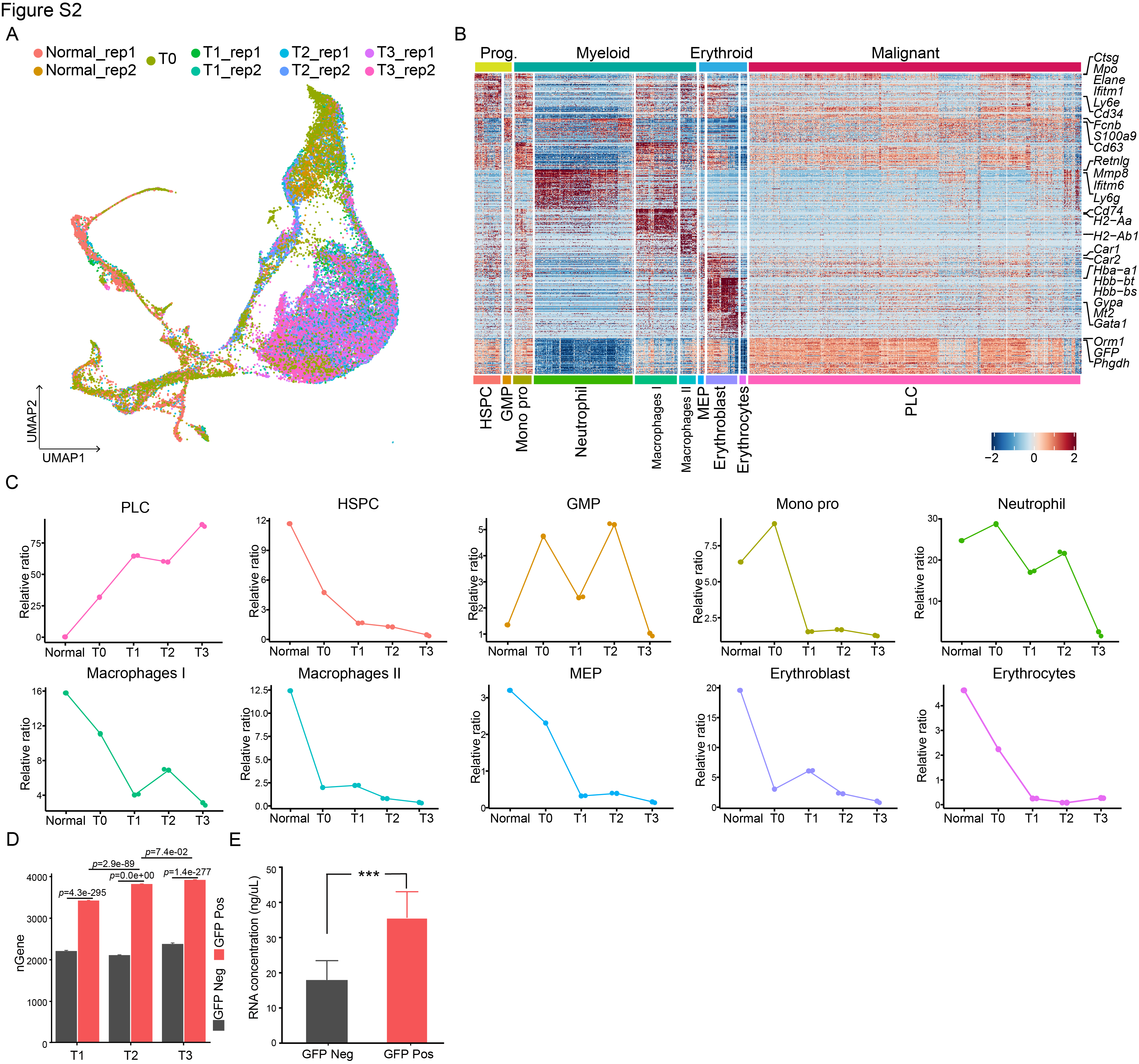

Supplement: S2 Fig — Related to Fig 1. (A) The UMAP plot of single cells for all 9 samples at 5 time points, colored by samples. (B) The heatmap showing the expression levels of cell type-specific genes among all cells, and classic markers were labeled on the right. (C) The line charts showing the dynamics percentage of each cell type during leukemogenesis. (D) Bar graph showing the number of genes in 3 times point; p values were calculated by Wilcoxon signed-rank test. (E) Bar graph showing the RNA concentration of Myc-GFP-positive cells and Myc-GFP-negative cells at T1 time point. ***p < 0.001, p values were calculated using an unpaired parametric t test. The underlying data for S2B–S2E Fig can be found in S1 Data. (TIF) [file pbio.3002088.s002.tif]

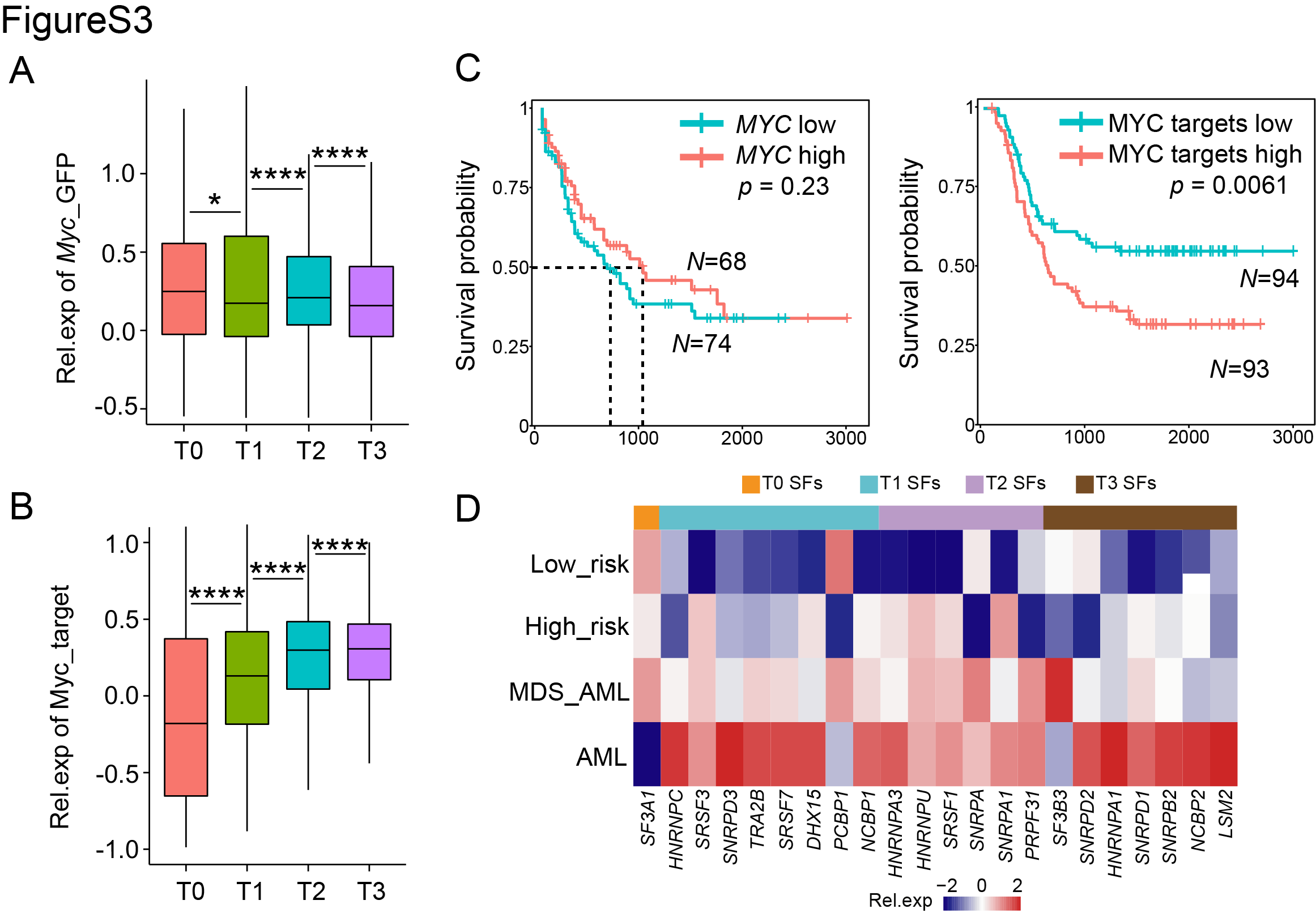

Supplement: S3 Fig — Related to Fig 2. (A) The box plot showing the expression levels of Myc at each time point during leukemogenesis; p values were calculated by Wilcoxon signed-rank test. (B) The box plot showing the expression levels of Myc_target at each time point during leukemogenesis; p values were calculated by Wilcoxon signed-rank test. (C) The Kaplan–Meier survival curves of TARGET-AML patients with low or high MYC/MYC targets; p value was calculated by log-rank test. (D) The heatmap showing the relative expression levels of 4 subtypes of MYC targets involved in splicing factors (columns), during leukemogenesis (rows), in human AML. The underlying data for S3A–S3C Fig can be found in S1 Data. (TIF) [file pbio.3002088.s003.tif]

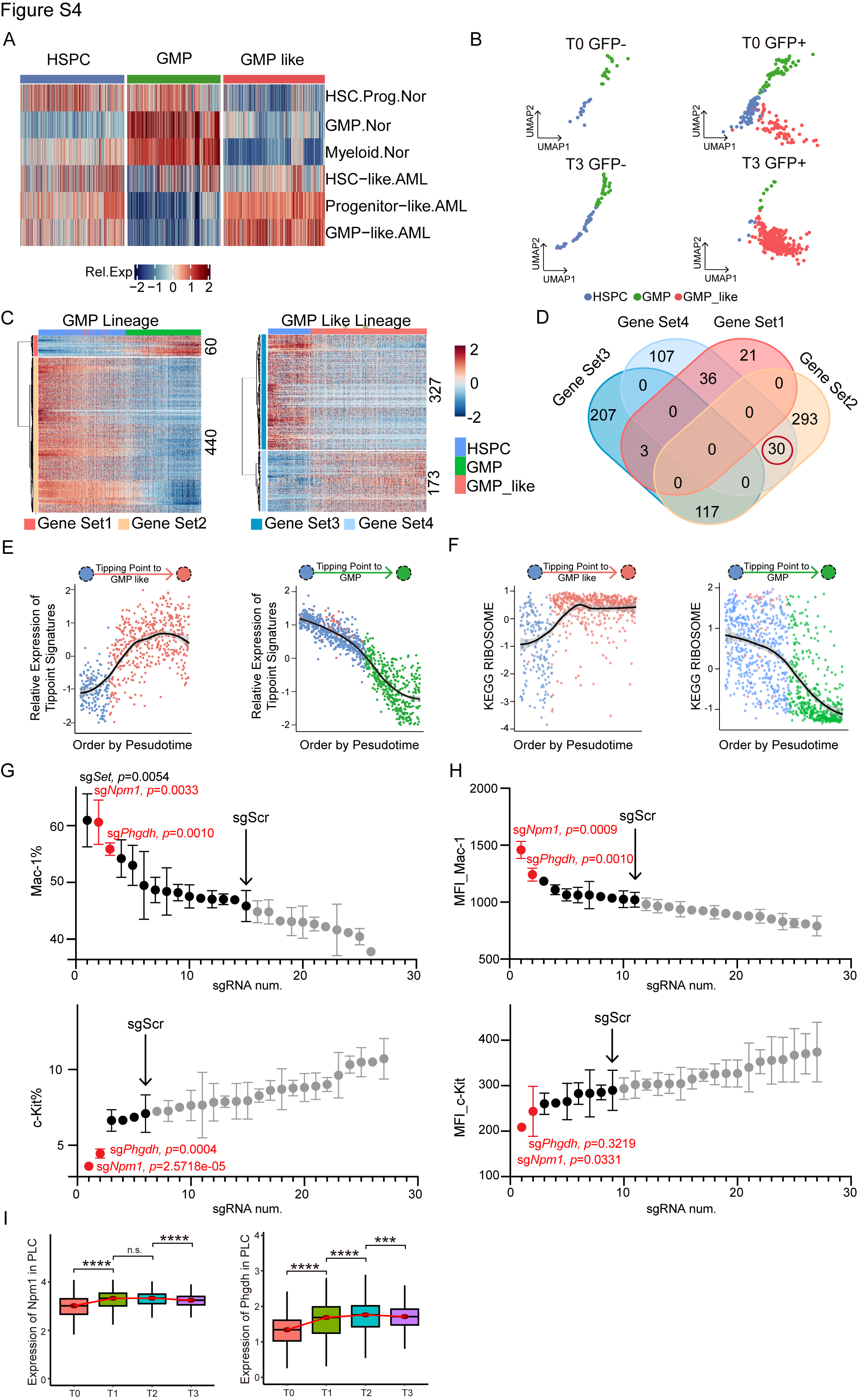

Supplement: S4 Fig — Related to Fig 3. (A) The heatmap showing the expression levels of normal HSC progenitor, GMP, myeloid and AML HSC-like, progenitor-like, GMP-like makers in the Galen and colleagues, Cell 2019 (rows) in tipping point HSPC, GMP, and GMP-like cells (columns) [18]. (B) The UMAP plot of single cells at tipping point stages split by samples and colored by cell annotation. (C) Heatmaps showing gene expression dynamics over pseudo time in GMP lineage (left) and GMP-like (right) lineage. Genes (row) are clustered and cells (column) are ordered according to the pseudo time. Gene set1: up-regulate genes in GMP lineage, Gene set2: down-regulate genes in GMP lineage, Gene set3: down-regulate genes in GMP-like lineage, Gene set4: up-regulate genes in GMP-like lineage. (D) Venn diagram showing the overlap among gene set1, gene set2, gene set3, and gene set4. (E) The tipping point signature expression trends along GMP lineage (left) and GMP-like lineage (right). Single cell colored by cluster annotation. (F) The Ribosome signature expression trends along GMP lineage (left) and GMP-like lineage (right). (G) Relative proportions of Mac-1+ (top) and of c-Kit+ (bottom) cell populations, measured by flow cytometry, in HSPCs infected with CRISPRs targeting tipping point genes or scramble. (H) Relative MFIs of Mac-1 (top) and of c-Kit (bottom) staining in HSPCs infected with CRISPRs targeting tipping point genes or scramble. (I) The box plot showing the expression levels of Npm1/Phgdh in PLCs at each time point during leukemogenesis. ****p.adj < 0.0001.p values were calculated by Wilcoxon test. The underlying data for S4G–S4I Fig can be found in S1 Data. (TIF) [file pbio.3002088.s004.tif]

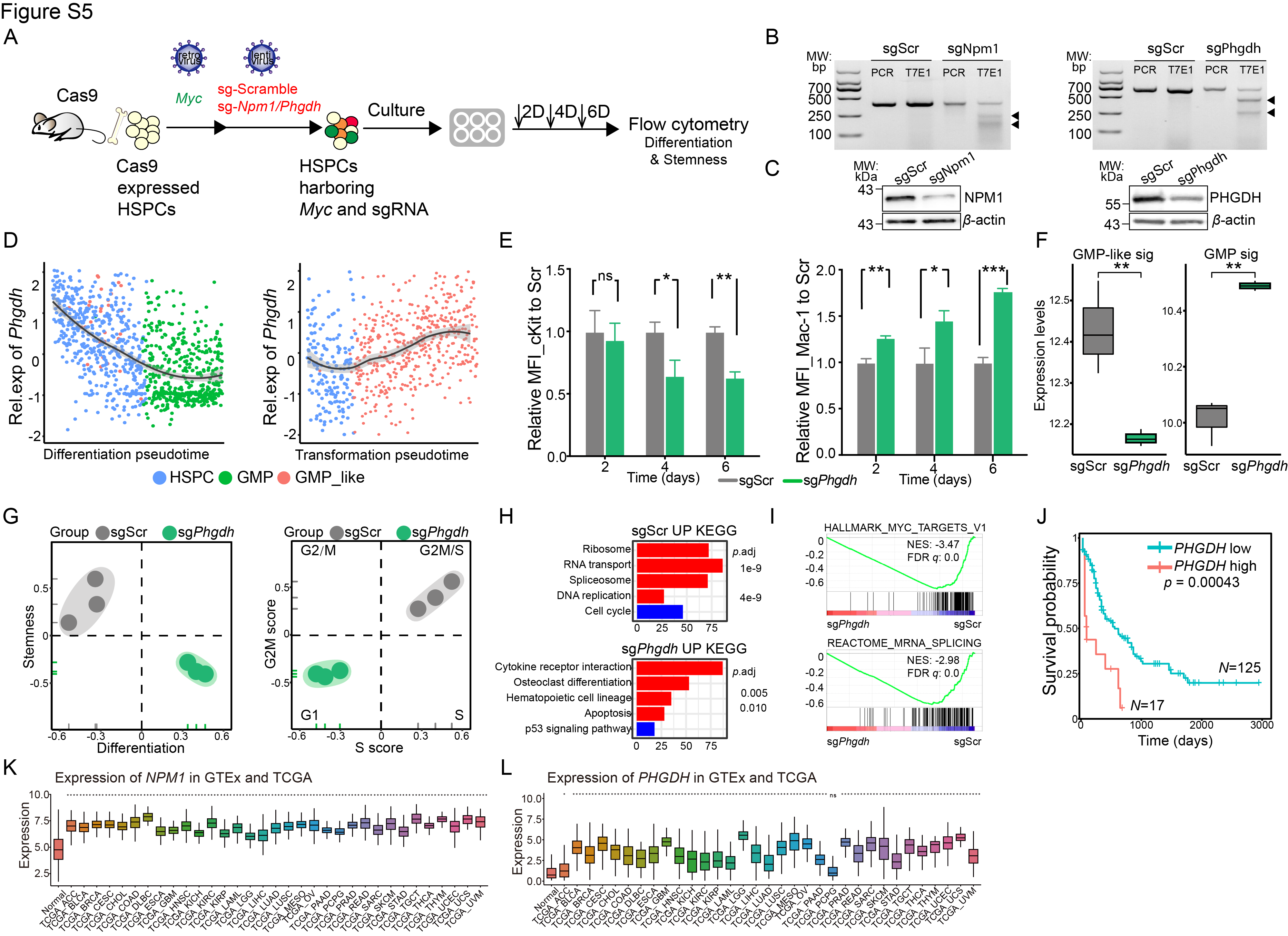

Supplement: S5 Fig — Related to Fig 4. (A) The schematic diagram showing the in vitro flow assay in Cas9 expressing HSPCs infected with Myc-GFP and sgRNA-mCherry. (B) T7 endonuclease I assay showed the sgNpm1/Phgdh efficiency in c-Kit+ cells derived from Cas9 expressing mouse. (C) Western blotting showing the protein levels of NPM1 and PHGDH in HSPCs edited with sgScr or sgNpm1/Phgdh. (D) The relative expressions of Phgdh along with the differentiation (top) and transforming (bottom) trajectories. (E) The bar graphs showed the MFI of stem cells (left) and differentiated cells (right) at 2 days, 4 days, and 6 days, in sgPhgdh samples, compared to scramble samples. *p < 0.05, **p < 0.01, ***p < 0.001, p values were calculated using an unpaired parametric t test. (F) The box plot showing the expression levels of GMP-like signature (left) and GMP signature (right) in sgPhgdh and sgScramble samples. *p < 0.05, **p < 0.05, p values were calculated by likelihood ratio test. (G) The scatter plot showing differentiation/stemness signature scores (left) and G2M/S scores (right) in sgPhgdh and sgScramble samples. Measured by RNA-seq. (H) The KEGG pathways enriched in sgScramble samples, compared to sgPhgdh cells (top). The KEGG pathways enriched in sgPhgdh samples, compared to sgScramble cells (bottom). (I) GSEA showing the negative enrichment of HALLMARK_MYC_TARGETS_V1 and HALLMARK_MRNA_SPLICING in sgPhgdh cells, comparing to sgSCr cells. (J) Kaplan–Meier curve showing the survival of AML patients in TCGA stratified by the expression of PHGDH. P value was calculated by log-rank test. (K) The box plots showing the expression levels of NPM1 in the normal GTEx samples and TCGA cohorts. *p.adj < 0.05, ****p.adj < 0.0001, n.s. not significant, p values were calculated by Wilcoxon test. (L) The box plots showing the expression levels of PHGDH in the normal GTEx samples and TCGA cohorts. *p.adj < 0.05, ****p.adj < 0.0001, n.s. not significant, p values were calculated by Wilcoxon test. The underlying da [file pbio.3002088.s005.tif]

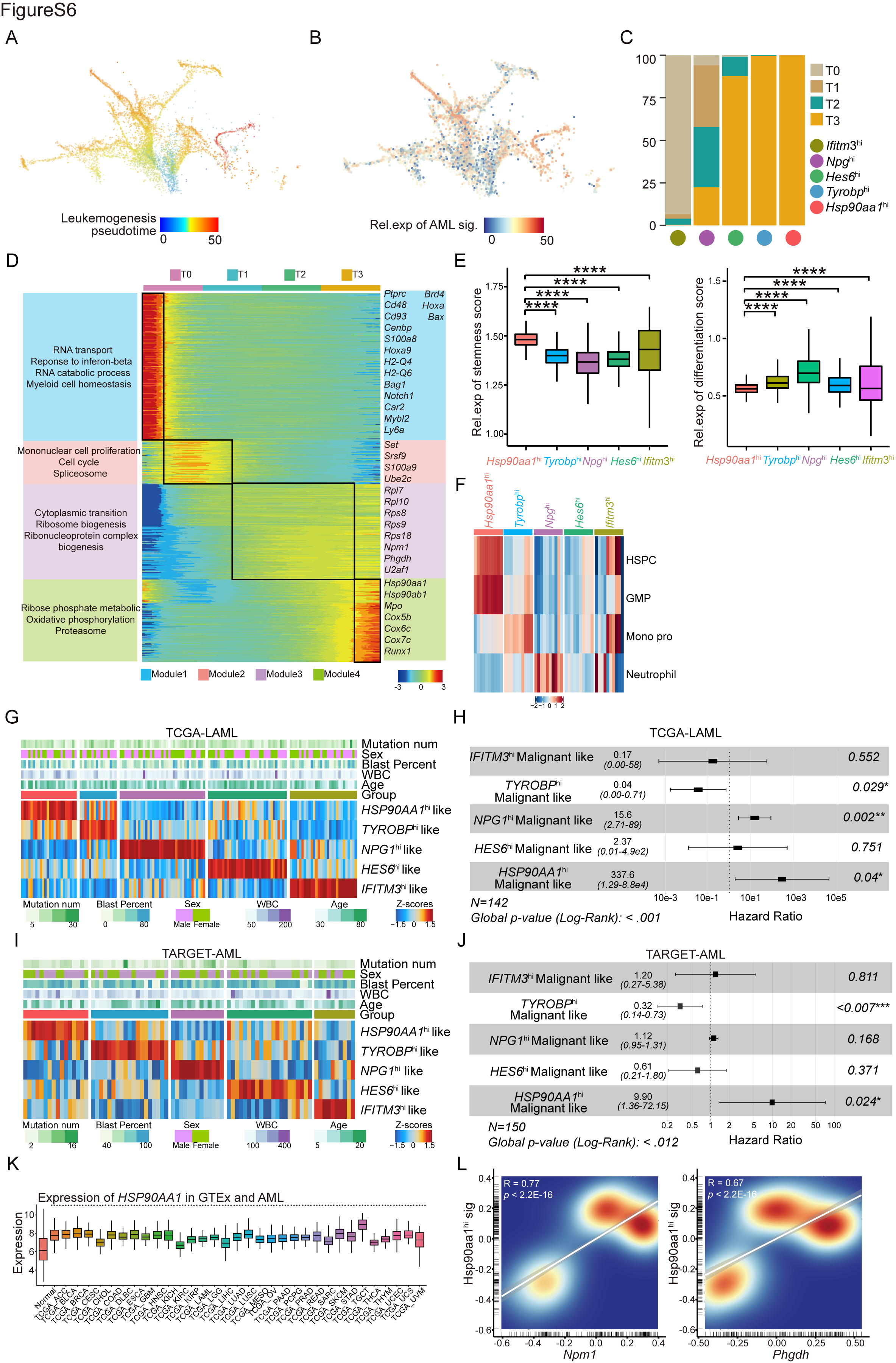

Supplement: S6 Fig — Related to Fig 5. (A, B) The force-directed layout maps showing leukemogenesis trajectories, colored by pseudo time (A) and the expressions of human AML signature genes (B). (C) The bar graph showing the proportion of each cell type during leukemogenesis. (D) The heatmap showing the dynamics molecular features during leukemia progression. The key genes and top enriched gene sets were labeled. (E) The box plot showing the stemness scores and differentiation scores in PLCs. ****p.adj < 0.0001, p values were calculated by Wilcoxon test. (F) The heatmap showing the expression levels of normal cell subtypes’ signatures in PLCs. (G) Heatmap summarized the expression of 5 malignant cell subtypes from T3 in TCGA-LAML database annotated by clinical information including mutation counts, sex, blast percentages, WBC, diagnosis ages, FAB, and subgroups of patients. (H) Hazard ratio calculated using the expression of 5 malignant cell subtypes from T3 in TCGA-LAML database. Hazard ratios >1 indicate an increased risk of dying. While hazard ratios <1 indicate a beneficial prognosis for the patient; p values of each individual factor based on the multivariate analysis is depicted on the right of the figure with the values: *p < 0.05, **p < 0.01, ***p < 0.001. (I) Heatmap summarized the expression of 5 malignant cell subtypes in TARGET-AML database annotated by clinical information including mutation counts, sex, blast percentages, WBC, diagnosis ages, FAB, and subgroups of patients. (J) Hazard ratio calculated using the expression of 5 malignant cell subtypes from T3 in TARGET-AML database. Hazard ratios >1 indicate an increased risk of dying. While hazard ratios <1 indicate a beneficial prognosis for the patient; p values of each individual factor based on the multivariate analysis is depicted on the right of the figure with the values: *p < 0.05, **p < 0.01, ***p < 0.001. (K) The box plots showing the expression levels of HSP90AA1 in the normal GTEx samples and TCGA cohorts. *p.a [file pbio.3002088.s006.tif]

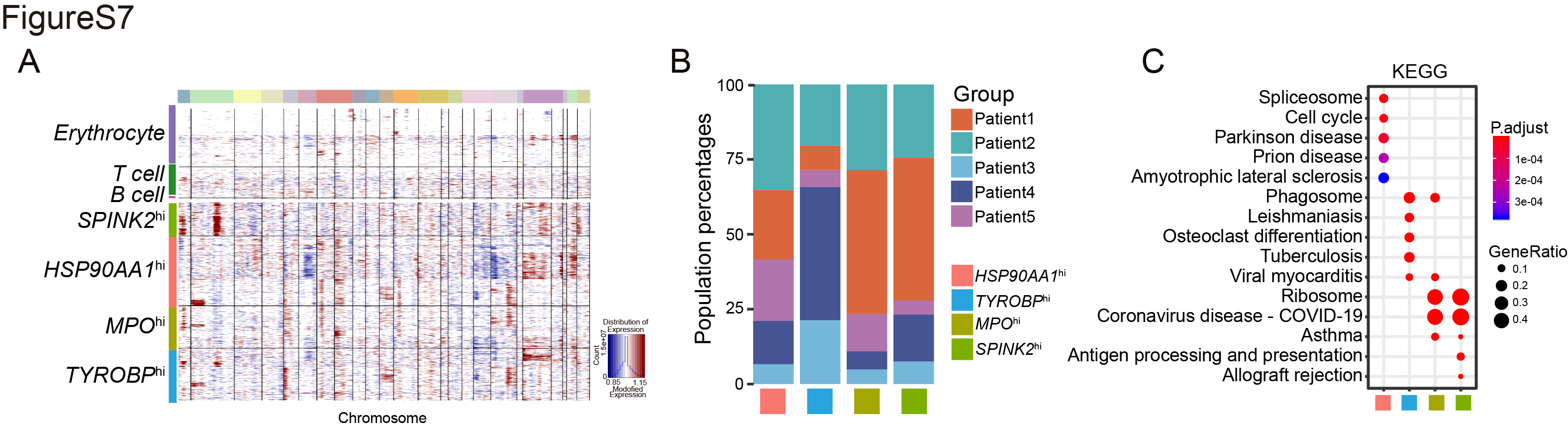

Supplement: S7 Fig — Related to Fig 5. (A) The heatmaps showed the results of inferCNV with hierarchical clustering in human AML. (B) The bar graph showing the proportion of 5 AML patients in each leukemia subtypes. (C) Top enriched KEGG pathways for differentially expressed genes in malignant cells. Node size represents gene ratio; node color represents p.adjust. p.adjust, adjusted p value. (TIF) [file pbio.3002088.s007.tif]

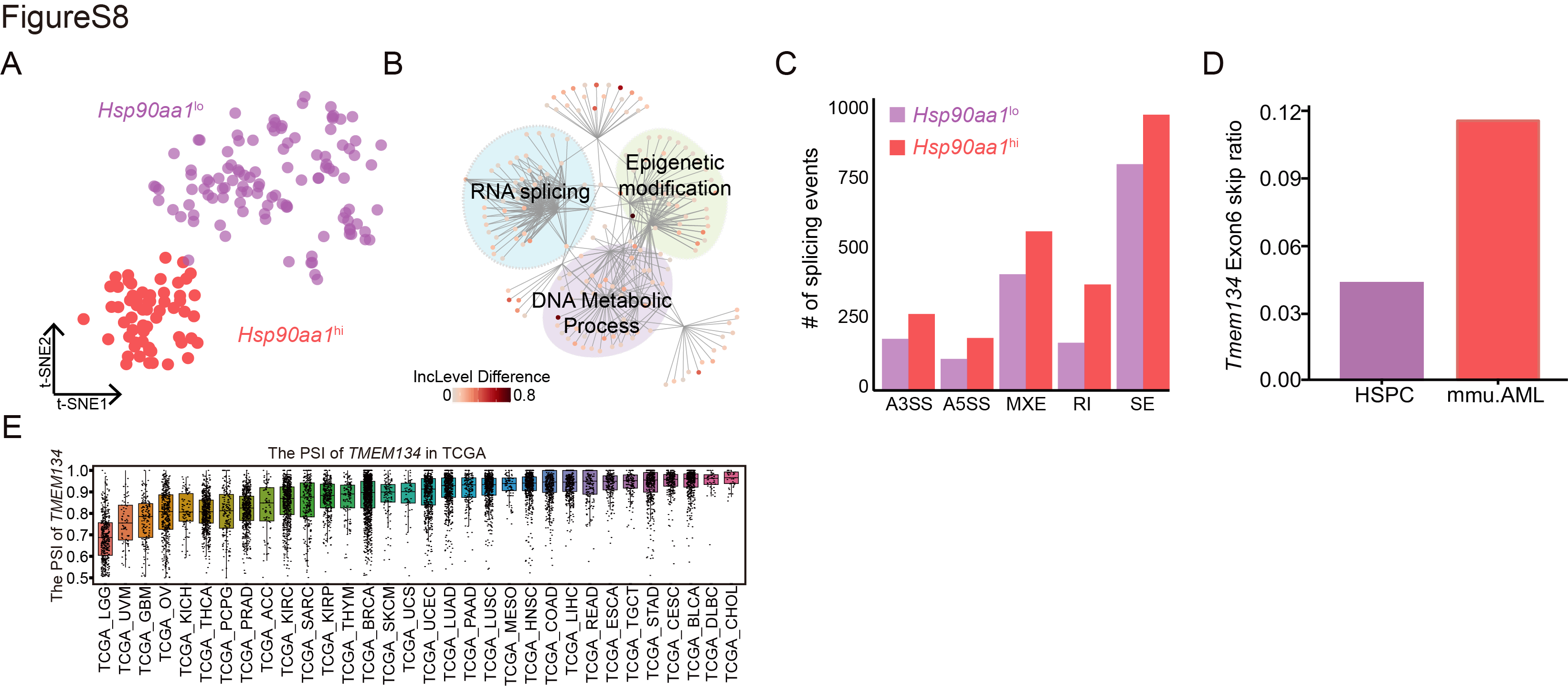

Supplement: S8 Fig — Related to Fig 6. (A) The t-SNE plot of T3 Myc-GFP-positive cells analyzed by Smart-seq2, colored by cell types. (B) The regulatory networks of signature genes in Hsp90aa1hi cells. (C) The bar graph showing the numbers of differentially alternative splicing events between Hsp90aa1lo and Hsp90aa1hi cells. (D) The bar graph showing the skipping ratio of Tmem134 exon6 in HSPC and AML. (E) The PSI values of exon6 skipping of TMEM134 in TCGA pan-cancer samples. The underlying data for S8C–S8E Fig can be found in S1 Data. (TIF) [file pbio.3002088.s008.tif]

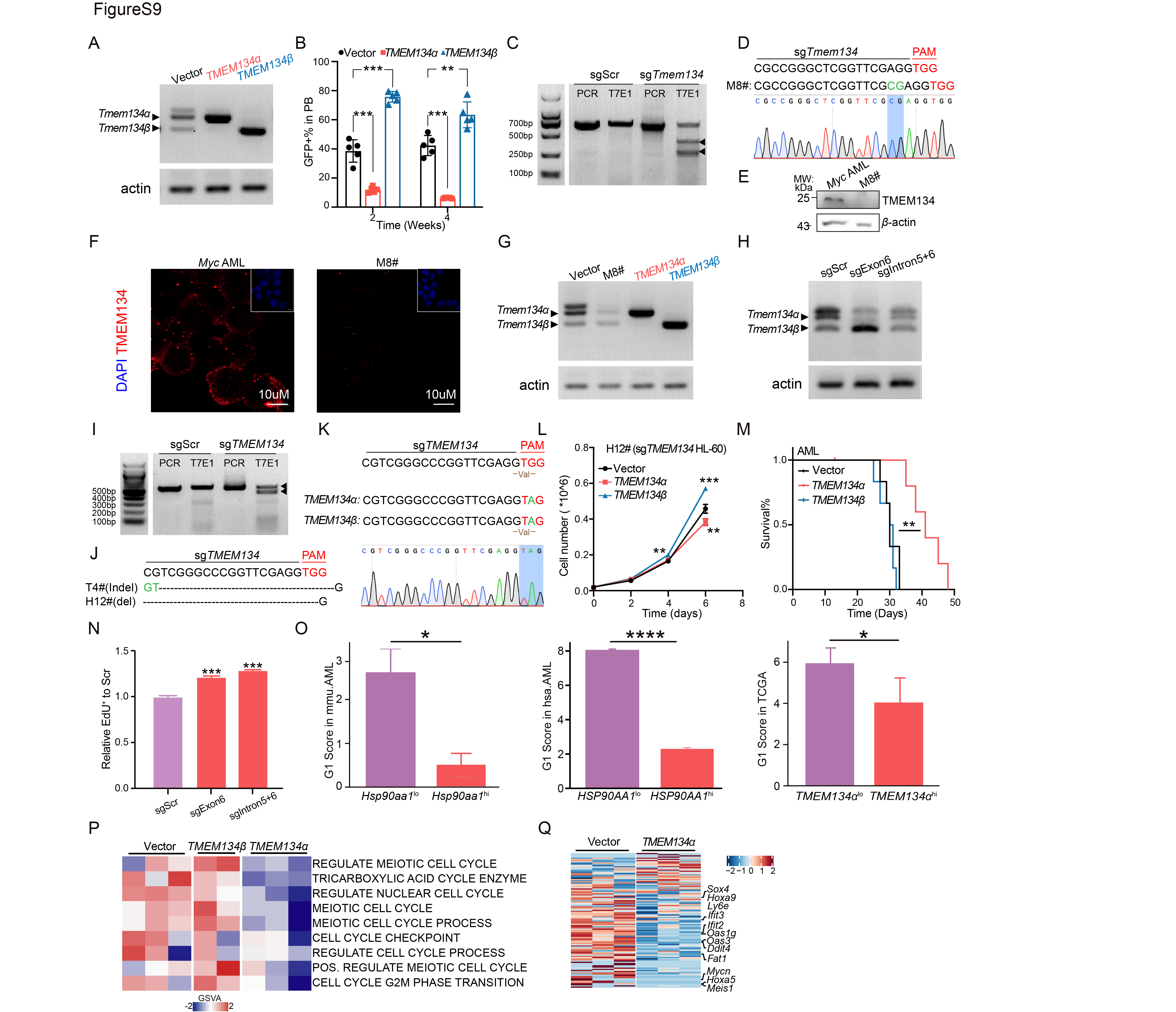

Supplement: S9 Fig — Related to Fig 6. (A) Semiquantitative PCR showed the relative mRNA expression of TMEM134α or TMEM134β in c-Kit+ HSPCs harboring vector, TMEM134α and TMEM134β. (B) Percentage of GFP-positive cells in peripheral blood collected from recipient mice with vector, TMEM134α and TMEM134β after 2- or 4-week transplantation. (C) T7 endonuclease I assay showed sgTmem134 efficiency in Myc-induced murine AML cells. (D) Sequence alignment result showing 2 bp insertion in one of the Tmem134-/- AML clones. (E) Western blotting showing of TMEM134 protein levels in Tmem134 wild-type (Myc AML) or knockout leukemic cells (M8#). (F) Immunofluorescence staining of TMEM134 protein in Tmem134 wild-type (Myc AML) leukemic cells or M8#. (G) Semiquantitative PCR showed the relative mRNA expression of TMEM134α or TMEM134β in endogenous Tmem134 KO mouse AML cells M8# harboring vector, TMEM134α and TMEM134β. (H) Semiquantitative PCR showed the relative mRNA expression of Tmem134α or Tmem134β in Myc-induced leukemic cells harboring sgScramble, sgExon6, or sgIntron5+6. (I) T7 endonuclease I assay showed sgTMEM134 efficiency in THP-1 cell line. (J) Sequence alignment results showing 2 bp insertion and 21 bp deletion in one of the Tmem134-/- THP-1 clones and 71 bp deletion in one of the Tmem134-/- HL-60 clones. (K) The sequence of synonymous mutated (PAM site) TMEM134α/β cDNA rescued in Tmem134 KO cell lines. (L) The growth curves of endogenous TMEM134 KO HL-60 cells with overexpressing vector, TMEM134α or TMEM134β at the indicated time points. **p < 0.01, ***p < 0.001. P values were calculated by unpaired parametric t test. (M) The Kaplan–Meier survival curves of recipient mice transplanted with leukemic cells overexpressing vector, TMEM134α or TMEM134β. **p < 0.01(log-rank test). (N) The bar graph showing the proliferative ability of Myc-GFP AML cells transduced with sgScramble, sgExon6, or sgIntron5+6, with EdU incorporation assay. ***p < 0.001, p values were calculated using an unpaired paramet [file pbio.3002088.s009.tif]

Fig 6D

Fig 6E

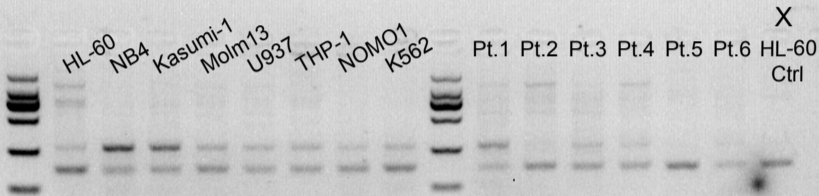

Fig S5B

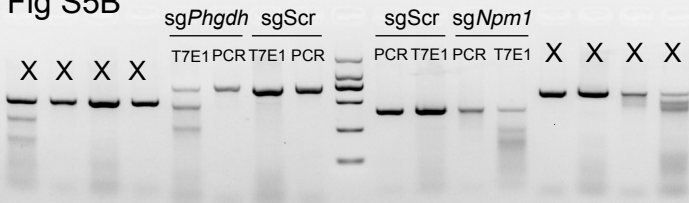

Fig S5C

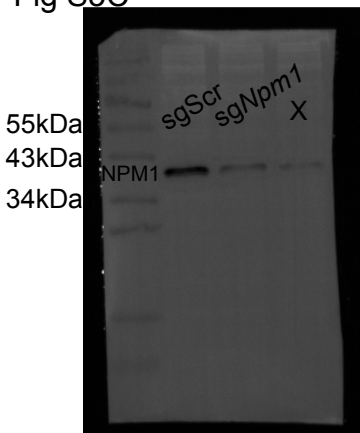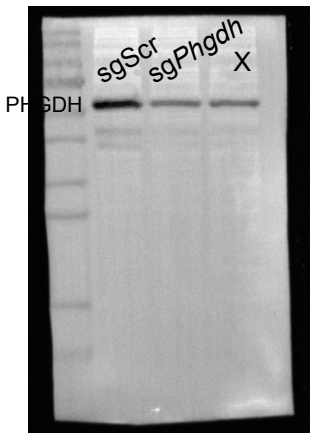

ACTIN

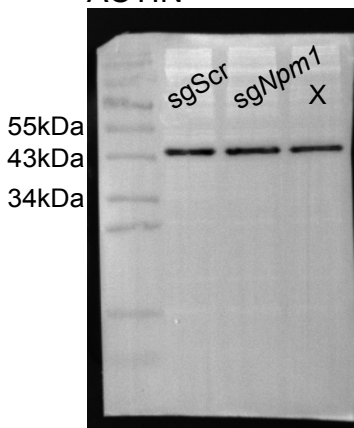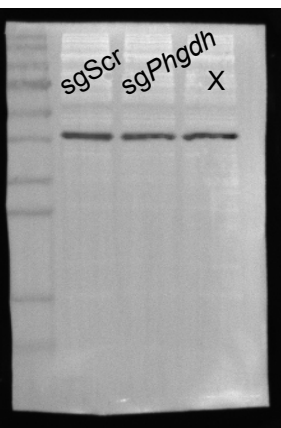

Fig S9C

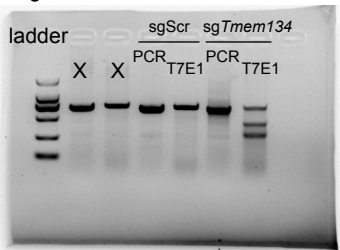

Fig S9I

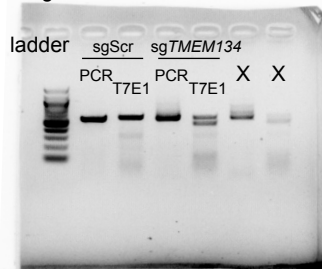

Fig S9H

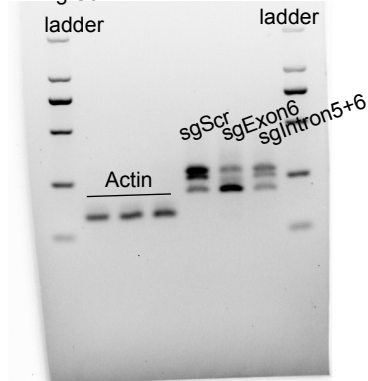

Fig S9A/G

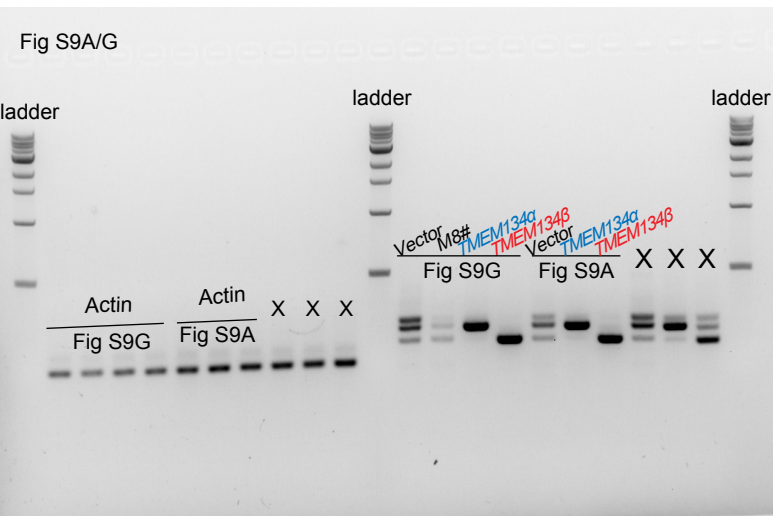

Fig S9E

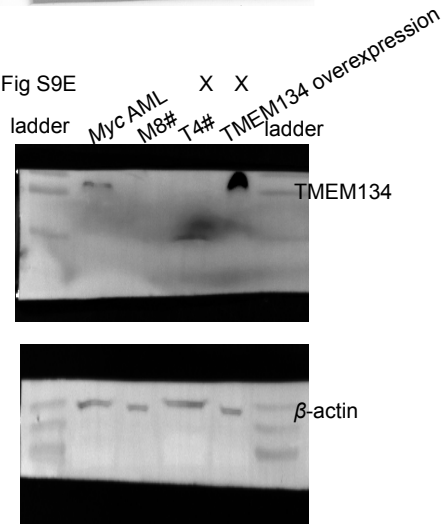

Supplement: S1 Raw Images — (PDF) [file pbio.3002088.s014.pdf]
